# Ten quick tips for teaching with participatory live coding

**DOI:** 10.1371/journal.pcbi.1008090

**Published:** 2020-09-10

**Authors:** Alexander Nederbragt, Rayna Michelle Harris, Alison Presmanes Hill, Greg Wilson

**Affiliations:** 1 Department of Biosciences, University of Oslo, Oslo, Norway; 2 Department of Informatics, University of Oslo, Oslo, Norway; 3 Department of Neurobiology, Physiology and Behavior, University of California, Davis, California, United States of America; 4 RStudio, PBC, Boston, Massachusetts, United States of America; University of Toronto, CANADA

## Introduction

### What is participatory live coding?

Participatory live coding is a technique in which a teacher or instructor writes and narrates code out loud as they teach and invites learners to join them by writing and executing the same code. Learners watch as an instructor writes code live in real time, typically via 1 or more projector screens that show the same screen as the instructor sees. Instructors also read out loud what they type, explaining the different elements and principles that are relevant for learners to understand the code. At the same time, each learner is invited to copy and execute the exact code or commands that are being written on their own work station. Learners thus “code-along” with the instructor. There are frequent, often short, exercises, in which learners are asked to solve a small relevant problem on their own.

This approach aims to be an improvement on teaching programming through lecturing showing static code or relying on learners reading a textbook or compendium. What is taught is immediately applied rather than just shown on a slide or on paper: It embodies the “I do, we do, you do” approach to knowledge transfer that is used both formally and informally to teach everything from laboratory bench skills to grant writing [1].

It also slows the instructor down, giving learners more time to actively engage with the material before moving on to the next concept. Importantly, the thought process behind coding can also be made explicit. Learner’s questions can immediately be answered and misconceptions corrected by coding them. Exercises enable immediate practice using the material.

Crucially, the technique also allows for teaching handling of mistakes. Beyond deliberately introducing mistakes during the live coding, instructors will often make unplanned mistakes. Novice learners are likely to make many such mistakes themselves, and diagnosing and solving mistakes is an integral aspect of learning programming.

The participatory aspect engages learners, which helps them become active practitioners rather than passive observers of the programming process. Participatory live coding is most beneficial for novices who are unfamiliar with the tools. More experienced learners may gain enough by listening passively or engaging with the material and classroom differently.

Participatory live coding for teaching programming should not be confused with live coding used to demonstrate software (for example, at a conference, with an audience passively observing) or live streaming programming [2] or live coding used as a form of performing art (e.g., while creating computer music [3]). A video recording demonstrating the participatory live coding technique can be found here: https://vimeo.com/139316669.

### Who uses it?

Participatory live coding is the main teaching method in workshops organized by the global nonprofit called The Carpentries (https://carpentries.org), the umbrella organization for Software Carpentry, Data Carpentry, and Library Carpentry [4]. The Carpentries teaches participatory live coding explicitly as a technique when training new instructors [5]. Increasingly, university courses involving the teaching of programming or related techniques employ the method (https://lexnederbragt.com/bios1100)[6].

There is a limited body of research on the effectiveness of live coding in programming education. Most studies focus on nonparticipatory live coding, demonstrating the programming process and contrasting this with the use of static code on slides (see [7] and references therein, as well as [8]). So far, these studies show that live coding is as good as, if not better than, using static code examples [7,9] and, thus, is a recommended approach for teaching programming [10,11].

The ten quick tips described below are aimed at those interested in applying participatory live coding to their own teaching. They are meant to complement the “Ten quick tips for teaching programming” [10].

## Tip 1: Go slowly

When using participatory live coding, you need to teach and program at a pace that allows learners to follow along and not get left behind. To do this, you will need to go slower than if you were coding alone. Say out loud what you are doing while you do it for every command you type, every word of code you write, and every menu item or website button you click. Then, point to the command and its output on the screen and go through it a second time. This allows learners to catch up and check their understanding: If they are watching you type and trying to type the same code in themselves, they do not have time to think about what they are doing. As you go through it a second time, they can check their understanding and correct any small typos they may have made. This is particularly important for learners who may not be fluent in the language of instruction or who may have hearing, vision, or mobility impairments.

If the output of your command or code makes what you just typed disappear from view, scroll back up so learners can see it again.

Asking your learners to copy and paste code or commands from your lesson material has the danger of you going too fast without explaining the thought process behind the code. If your current practice involves copying and pasting, try transitioning to a technique where you, the instructor, type everything in the console or editor, building the scripts one command at a time with the learners. Learners can copy and paste commands from the resources provided if they need to catch up or are practicing asynchronously with an online tutorial.

## Tip 2: Mirror your learner’s environment

If learners have to work in a different environment than you, mental effort is added that does not contribute to learning. Cognitive psychological theory calls this “extraneous cognitive load” [12]. Try to create an environment that is as similar as possible to what your learners have. For example, you may have personalized your environment with a very simple or rather fancy Unix prompt, color schemes for your development environment, keyboard shortcuts, etc. Your learners usually will not have made such modifications.

Similarly, avoid using keyboard shortcuts because these will hide the action(s) you are performing and learners may not know about them. If you must use them, use a keystroke visualizer tool or your computer’s accessibility tools to echo keystrokes to the screen.

Some instructors create a separate “bare-bone” user (login) account on their laptop or a separate “teaching-only” account on the service being taught (e.g., Github). It could be a benefit if both instructor and learners use the exact same software (terminal or code development environment); however, this may incur some effort to get the software installed on the computers that learners use. Using a cloud-based solution is an alternative to ensure all involved have the exact same setup during the teaching.

## Tip 3: Be seen and heard

As learners are coding along, it is important they clearly see and hear what you are doing. If you are physically able to stand up for the duration of your class, do it while you are teaching. When you sit down, you may appear hidden for those sitting in the back rows. Standing makes the experience more interactive, less monotonous, and draws the learners’ attention away from their screens to you, which helps getting the point you are making across. Standing also encourages you to look at your audience rather than your screen: If you are sitting, you are likely to do what you normally do when you are sitting, which is look at your own computer. It helps to have a high table, standing desk, or lectern so you can have your laptop at a comfortable height for typing.

Regardless of whether you are standing or sitting, consider breaking up the teaching with some movement. For example, you could walk to the screen to point something out or draw something on the whiteboard (see Tip 6 below).

Even though you may have a good voice and know how to use it well, it may be an advantage to use a microphone, especially if the room is equipped with one. You will tire your voice less, and you increase the chance of people with hearing difficulties being able to follow the teaching.

## Tip 4: Use the screen(s) wisely

When live coding, it is even more important than when presenting slides that all learners can see the relevant portions of the screen because they may want to copy exactly what you have typed or see what you are pointing to. Use a big font and maximize the window. Note that with a large font, you may have fewer columns and rows than you are used to, so you should design examples with this in mind (or at least test them). A black font on a white background works better than a light font on a dark background. When the bottom of the projector screen is at the same height or below the heads of the learners, people in the back will not be able to see the lower parts, so resize the window(s) you use on your computer (drawing up the bottom) to compensate.

Pay attention to the lighting: Avoid glare in rooms with digital screens and dim lights around projector screens.

If you can get a second projector, use it! It may require its own PC or laptop, so you may need to ask a helper to control it. You could for example display the live coding on one screen and use the second screen to present illustrations.

## Tip 5: Avoid distractions

Seeing notifications flash by on the screen distracts you as well as the learners and may even result in awkward situations when a message pops up you would rather not have others see. Turn off notifications on your laptop and phone, such as those from social media, email, etc. A difference between presenting a deck of slides and live coding is that you may end up sharing more of your computer during the latter. Close any applications that may cause distractions, and consider what desktop image and screensaver you use. Use printouts of the lesson material during teaching, or, alternatively, display them on a second device (tablet or laptop).

## Tip 6: Use illustrations—Even better, draw them

Lesson material often comes with illustrations, and these may help learners to understand the stages of the lesson and organize the material. What can work really well is when you as instructor generate the illustrations on the whiteboard as you progress through the material. This allows you to build up diagrams, making them increasingly complex in parallel with the material you are teaching. Presenting complementary information using visual and verbal representations helps learning (so-called “dual coding” [13]). Consider having learners draw or fill in diagrams themselves, from scratch or from fill-in-the-blank diagrams you have prepared in advance. Diagramming helps learners understand the material, makes for a more lively workshop (you will have to move between your computer and the whiteboard), and gathers the learners’ attention to you as well.

## Tip 7: Stick to the lesson material

When getting started with participatory live coding when teaching, it is advised to use a well-developed and tested lesson. Examples of such well-tested lessons specifically written for teaching using this technique are the collaboratively developed lessons from The Carpentries (https://carpentries.org/workshops-curricula). Practicing teaching your lesson material in advance is important because the participatory live coding technique can be more demanding, especially when you are doing it for the first time. Add notes to your printouts of the lesson material, or have them easily available on the second device (tablet or laptop), if you use one. It may be tempting to deviate from your material because you would like to show a neat trick or demonstrate some alternative way of doing something, but there is always a fair chance you will run into something unexpected that you then have to explain. It is, thus, advised not to improvise until you are familiar enough with the material and want to explain something with a low risk of failure. Consider the use of a timer for exercises: They help keep yourself honest when you tell learners they have 5 minutes for an exercise. Sometimes a question or a “what if?” comes up that you would like to address but need some time to sort through. Collect these, for example, on sticky notes, or ask learners to add them to a shared online document that they all can edit. This way, you can think about these while learners are doing exercises and answer them afterwards. You then do not disrupt the flow of the lesson, while still showing that you take the learner’s questions seriously.

## Tip 8: Embrace your mistakes

No matter how well prepared you are, you will make mistakes: Typos are hard to avoid, you may overlook something from the lesson instructions, etc. Experiencing the instructor making mistakes allows learners to see how to diagnose and correct them as well as giving the learners permission to make and share theirs. This is a way of dealing with mistakes that is called “positive error framing,” and it has shown to be beneficial for learning [14]. So, do not fear making mistakes and turn them into a teachable moment: Novices are going to spend at least some of their time making similar mistakes, but how to deal with them is left out of most textbooks. For example, read out the error message, and explain how it told you what mistake you made. You can also involve the learners in the problem-solving by asking them what they think went wrong and how it can be fixed.

## Tip 9: Get real-time feedback and provide immediate help

It can be difficult while teaching to be sure all learners are following along and do not fall behind. One way to check with your learners is to give each learner 2 sticky notes of different colors, e.g., blue and yellow. These can be held up for voting, but their real use is as status flags. If someone has completed an exercise, they put the yellow sticky note on their laptop; if they run into a problem and need help, they put up the blue one. This is better than having people raise their hands because they can keep working while their flag is raised, while signaling to any available helpers who to go to. It is important to check with your learners if all of them can distinguish between the different colors of the sticky notes. For example, many people are red–green colorblind, and some people are blue–yellow colorblind. As an alternative, colorblind learners could mark the stickers by writing something onto them, e.g. “OK” and “Problem.”

Also, the use of sticky notes allows the instructor to quickly see from the front of the room what state the class is in. Sometimes a blue sticky note involves a technical problem that takes a bit more time to solve. To prevent this issue slowing down the whole class too much, use the occasion to take the small break you had planned to take a bit later, giving yourself or any helpers you may have time to fix the problem.

If learners are not putting up a sticky note when they are stuck, asking them to do pair programming during the live coding may help: 1 learner does the typing, while the other offers comments and suggestions, with frequent role switches. Learners may feel it easier to ask their partner, and if they are both stuck, the frustrated conversation between them is more likely to be noticed by the instructor or a helper than 1 person’s puzzled silence is.

If you can, have a good ratio between helpers and/or teaching assistants and learners. How many helpers you need and can have depends on the course or workshop, but around 1 helper for each 10 students is a good place to start. The main role for helpers is assuring learners do not fall behind due to, for example, technical issues. Helpers should keep an eye out for sticky notes indicating a learner signaling for help. A co-instructor can share the burden of teaching and responding to student needs because participatory live coding can be particularly tiring for students as well as instructors. While they are not teaching, a co-instructor can also keep an eye on the room and give the instructor some immediate feedback on the pace or other issues.

When you need to manage a diverse classroom in which you have a range of experience and expertise amongst your learners, you run the risk of more experienced learners becoming demotivated because they feel they are not learning anything new. You can recognize experienced learners by the questions they ask and the speed with which they complete the first few exercises. To keep them engaged, you could ask them to go and help someone who has a blue sticky note up. If they understand the problem, the other student gets feedback and help more quickly. If the more experienced learner does not understand the problem, it is a fresh challenge from which they will learn. More experienced learners can also be asked to do the bulk of notetaking in the shared online document, if one is being used.

## Tip 10: Turn learners into co-instructors

During participatory live coding, learners are actively coding along with the instructor. You can engage them even more in different ways. For example, have learners call out the next line of code that they think you as instructor should type next. It helps you to understand any misunderstandings learners might have as well as having them practice applying the material taught. You can also ask them to take notes collaboratively, using an online notetaking document that they all can edit. Having learners discuss and verbalize the material they just learned in their own words helps solidify their knowledge. When you need to manage a diverse classroom where you have a range of experience and expertise amongst your learners, asking the more experienced learners to contribute may also help keep them motivated.

## Conclusion

Participatory live coding is used successfully by thousands of instructors all over the world teaching programming, the use of the Unix shell, or version control. It is increasingly being used in undergraduate teaching. It takes some practice to get used to presenting material this way. But after a few tries, most people feel it becomes natural and rewarding as you interact with your learners in a wholly different way than when presenting slides.

Teaching is performance art and can be rather serious business. Do not let this scare you: It is okay to add an element of play, i.e., use humor and improvisation to liven up the teaching. How much you are able and willing to do this is really a matter of personality and taste as well as experience. It becomes easier when you are more familiar with the material, allowing you to relax more. Choose your words and actions wisely, though. Remember that you want the learners to have a welcoming experience and a positive learning environment: A misplaced joke can ruin things in an instance. Start small: Just saying “that was fun” after something worked well is a good start.

### Sources for these ten quick tips and further reading

These tips were developed in the context of Software Carpentry and a first edition appeared on their blog (https://software-carpentry.org/blog/2016/04/tips-tricks-live-coding.html). They have become part of The Carpentries instructor training materials (https://carpentries.github.io/instructor-training). These also use example videos contrasting live coding done poorly (https://youtu.be/bXxBeNkKmJE) and live coding done well (https://youtu.be/SkPmwe_WjeY). Finally, [15] has a section on live coding.
